# FGF21 controls hepatic lipid metabolism via sex-dependent interorgan crosstalk

**DOI:** 10.1172/jci.insight.155848

**Published:** 2022-10-10

**Authors:** Aki T. Chaffin, Karlton R. Larson, Kuei-Pin Huang, Chih-Ting Wu, Nadejda Godoroja, Yanbin Fang, Devi Jayakrishnan, Karla A. Soto Sauza, Landon C. Sims, Niloufar Mohajerani, Michael L. Goodson, Karen K. Ryan

**Affiliations:** 1 Department of Neurobiology, Physiology and Behavior, College of Biological Sciences, and; 2 Department of Anatomy, Physiology and Cell Biology, School of Veterinary Medicine, University of California, Davis, Davis, California, USA.

**Keywords:** Endocrinology, Hepatology, Growth factors, Obesity, Sex hormones

## Abstract

The liver regulates energy partitioning and use in a sex-dependent manner, coupling hepatic substrate availability to female reproductive status. Fibroblast growth factor 21 (FGF21) is a hepatokine produced in response to metabolic stress that adaptively directs systemic metabolism and substrate use to reduce hepatic lipid storage. Here we report that FGF21 altered hepatic transcriptional and metabolic responses, and reduced liver triglycerides, in a sex-dependent manner. FGF21 decreased hepatic triglycerides in obese male mice in a weight loss–independent manner; this was abrogated among female littermates. The effect of FGF21 on hepatosteatosis is thought to derive, in part, from increased adiponectin secretion. Accordingly, plasma adiponectin and its upstream adrenergic receptor → cAMP → exchange protein directly activated by cAMP signaling pathway was stimulated by FGF21 in males and inhibited in females. Both ovariectomized and reproductively senescent old females responded to FGF21 treatment by decreasing body weight, but liver triglycerides and adiponectin remained unchanged. Thus, the benefit of FGF21 treatment for improving hepatosteatosis depends on sex but not on a functional female reproductive system. Because FGF21 provides a downstream mechanism contributing to several metabolic interventions, and given its direct clinical importance, these findings may have broad implications for the targeted application of nutritional and pharmacological treatments for metabolic disease.

## Introduction

Male and female mammals experience divergent energetic requirements to ensure successful reproduction, resulting in a well-appreciated sex difference in the provisioning and utilization of fuel sources (1, 2). At rest, males tend to oxidize free fatty acids (FFAs) whereas females incorporate FFAs into triglycerides to be stored as fat to support their continued reproductive capacity in the face of limited food availability. During pregnancy and lactation, these energy stores are mobilized, which spares glucose and proteins for growth of the offspring (1). When individuals are subjected to complete starvation, females have a greater ratio of lipid to protein loss and are more likely to survive (3, 4). The liver plays a critical role in this sex-dependent control of substrate utilization, and recent evidence describes the coupling of hepatic lipid, carbohydrate, and amino acid availability to female reproductive status (5).

Fibroblast growth factor 21 (FGF21) is a protein hormone secreted from the liver and adipose tissue in response to nutritional and/or intracellular stress (6–9), and it acts both locally and via interorgan crosstalk to direct macronutrient intake and metabolism (10–12). In the adipose tissues, FGF21 signaling increases glucose uptake (13), increases mitochondrial biogenesis and oxidative metabolism (14, 15), and induces secretion of the hormone adiponectin (16, 17). In the liver, FGF21 decreases triglyceride content by reducing lipid uptake and de novo lipogenesis and by increasing hepatic fatty acid oxidation (18–20). Consequently, FGF21 is a promising therapeutic target for the treatment of fatty liver disease (21–23). The effects of FGF21 on hepatic lipid handling are recapitulated by pharmacological treatment with adiponectin (24, 25). Accordingly, interorgan crosstalk via adiponectin is thought to provide a key mechanism contributing to the beneficial effects of FGF21 in the liver (16, 17), including for hepatosteatosis, since FGF21 is ineffective in reducing liver triglycerides in adiponectin-null mice (17).

FGF21 is therefore well situated to direct the sex-dependent control of hepatic lipid handling. In agreement with this possibility, several recent studies report sex-dependent control of FGF21 secretion (26, 27). Moreover, we recently reported that several of the metabolic effects of dietary protein dilution, which are mediated by FGF21 (28–30), are sex dependent and that this sexual dimorphism requires the presence of an intact female reproductive system (31). In the present study we investigated the role of sex and reproductive status on the control of adiponectin secretion and liver triglycerides by FGF21.

## Results

### Effects of FGF21 on energy balance are sex dependent.

**Figure 1:**
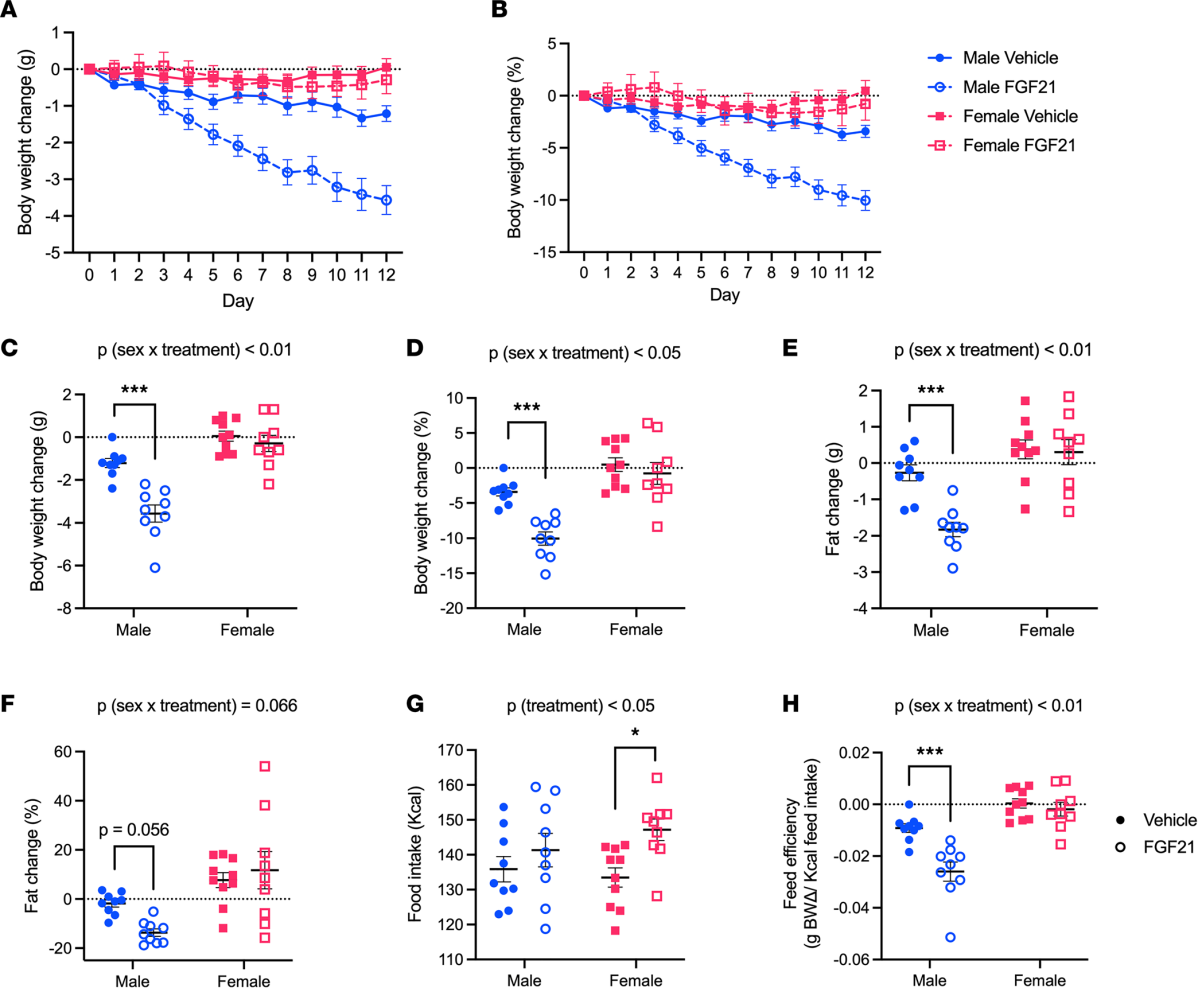
Effects of FGF21 on energy balance are sex dependent. FGF21 (0.1 mg/kg twice daily, i.p., 12 days) decreased body weight in DIO male but not female mice (**A**–**D**). This sex-dependent body weight loss was primarily derived from fat loss (**E** and **F**). FGF21 increased caloric intake in both males and females (**G**), while decreasing feed efficiency in males only (**H**). Analyses made by 2-way ANOVA, Tukey post hoc test, *P < 0.05, ***P < 0.001. Data are shown as mean ± SEM; *n* = 9–10 mice/group.

To determine the importance of sex as a variable affecting the control of energy balance by FGF21, we administered FGF21 by intraperitoneal (i.p.) injection to diet-induced obese (DIO) male and female C57BL/6J littermates for 12 days. FGF21-treated mice lost body weight in a sex-dependent manner [*P* (treatment × sex) < 0.05], affecting males (Tukey, *P* < 0.001) but not females. This was true whether weight change was expressed as an absolute value (g) or as a percentage of total starting body weight (Figure 1, A–D). As expected, body composition analysis (time domain NMR, Bruker) demonstrated the weight loss was derived primarily from fat [*P* (treatment × sex) < 0.01], likewise affecting males (Tukey, *P* < 0.001) but not females (Figure 1, E and F). Consistent with previous reports (11), FGF21-treated mice had greater caloric intake relative to controls, even as males lost weight. The effect of FGF21 to increase caloric intake occurred in both males and females [*P* (treatment) < 0.05], though it was perhaps more apparent among the female littermates (Tukey, *P* < 0.05) (Figure 1G). Accordingly, feed efficiency was decreased by FGF21 in males (Tukey, *P* < 0.001) but not female mice (Figure 1H).

### Effect of FGF21 on glucose tolerance is not dependent on sex.

**Figure 2:**
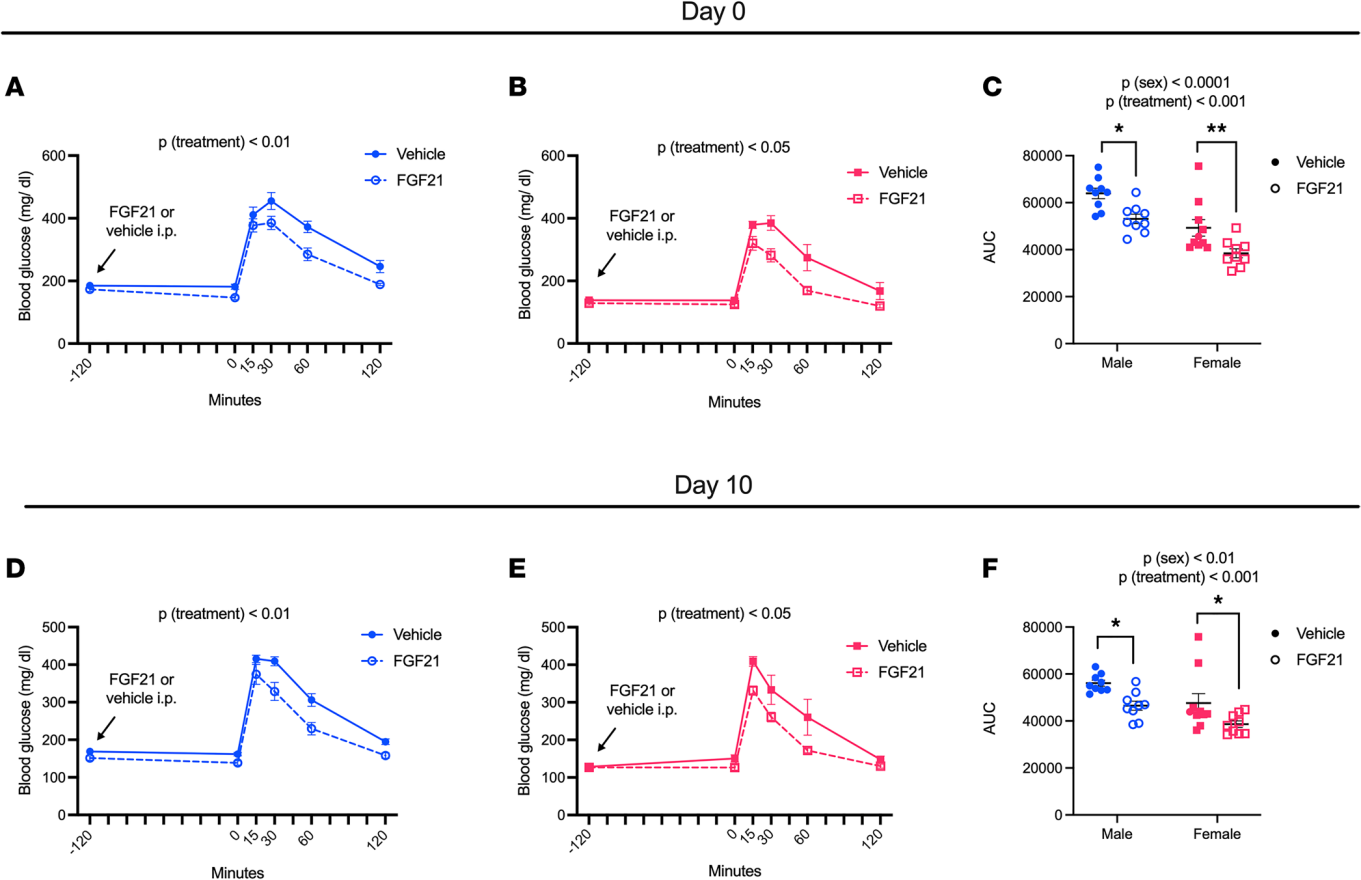
Effect of FGF21 on glucose tolerance is not dependent on sex. At 2 hours (**A**–**C**) and 10 days (**D**–**F**) following the initial dose of chronic FGF21 (see Figure 1), DIO male and female mice underwent glucose tolerance tests. Blood glucose was measured from the tail vein immediately following the morning dose of FGF21 or vehicle. Two hours later, blood glucose was measured immediately prior to a dextrose administration and at 15, 30, 60, and 120 minutes postinjection. FGF21 improved glucose tolerance in both males and females on day 0 (**A**–**C**) and on day 10 (**D**–**F**). Analyses made by repeated measures 2-way ANOVA (**A**, **B**, **D**, and **E**) and 2-way ANOVA (**C** and **F**), Tukey post hoc test, *P < 0.05, **P < 0.01. Data are shown as mean ± SEM; *n* = 9–10 mice/group.

To determine the importance of sex as a variable affecting the control of blood glucose by FGF21, we performed i.p. glucose tolerance tests immediately following the first dose of FGF21 or vehicle and again after 10 days of twice daily i.p. FGF21 injections. In agreement with the literature, FGF21 improved glucose tolerance as early as 2 hours following the initial dose, and this was true for both males [*P* (treatment) < 0.01] (Figure 2A) and females [*P* (treatment) < 0.05] (Figure 2B). When comparing the area under the curve, we observed a main effect of sex (*P* < 0.0001) and treatment (*P* < 0.001), and there was no interaction between sex and treatment (Figure 2C). Likewise, on day 10, FGF21 improved glucose tolerance in both males [*P* (treatment) < 0.01] (Figure 2D) and females [*P* (treatment) < 0.05] (Figure 2E). Again, we observed a main effect of sex (*P* < 0.01) and treatment (*P* < 0.001) but no interaction between sex and treatment (Figure 2F). Thus, FGF21 improved glucose control in both males and females, despite divergent effects on body weight in this study.

### Effects of FGF21 on liver triglycerides are sex dependent, and this does not depend on body weight loss.

**Figure 3:**
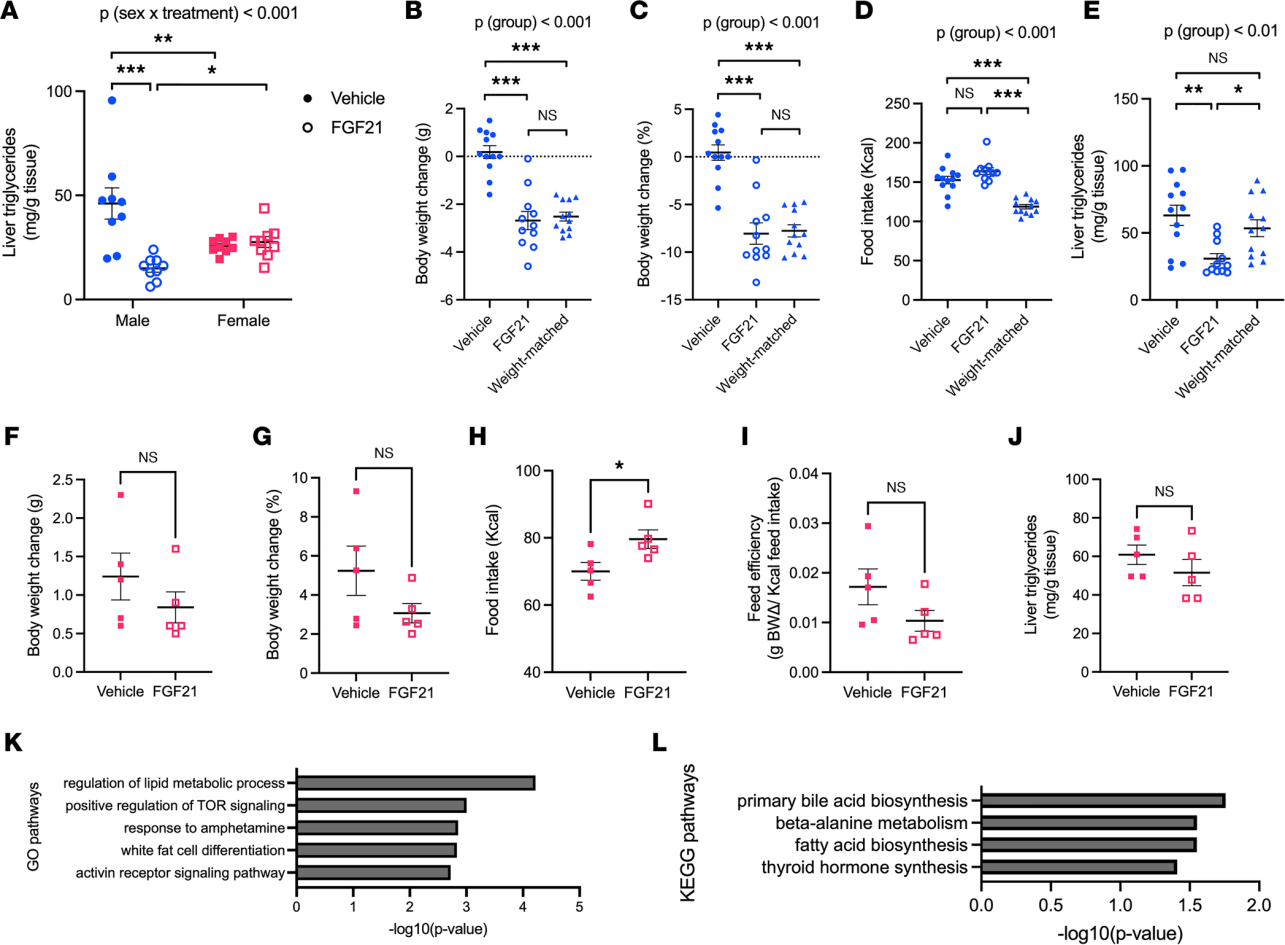
Effects of FGF21 on liver triglycerides are sex dependent, and this does not depend on body weight loss. FGF21 (0.1 mg/kg twice daily, i.p., 12 days) decreased liver triglycerides in DIO male but not female mice (**A**). In a separate experiment, DIO male mice were assigned to ad libitum–fed + vehicle, ad libitum–fed + FGF21, or a weight-matched group, in which caloric intake was restricted (**D**) to match the weight loss observed by FGF21-treated mice (**B** and **C**). Compared with vehicle-treated mice, FGF21 treatment decreased liver triglycerides, but weight matching did not, despite weight-matched mice losing the same amount of weight as FGF21-treated mice (**E**). In another experiment, a higher dose of FGF21 (1 mg/kg once daily, sc, 5 days) administered to DIO female mice again increased food intake (**H**) but did not decrease body weight (**F** and **G**), feed efficiency (**I**), or liver triglycerides (**J**). Next, to identify metabolic pathways contributing to the sex-dependent change in liver triglycerides (**A**), transcriptomic and metabolomics analyses were conducted on livers after acute FGF21 administration (2 doses of 0.1 mg/kg, i.p.). We identified differential expression results for the treatment × sex interaction term, revealing 173 significantly enriched GO pathways from transcriptomic analyses (top 5 shown in **K**) and 4 significantly enriched KEGG pathways from metabolomic analyses (**L**). Analyses made by 2-way ANOVA (**A**, *n* = 9 mice/group) or 1-way ANOVA (**B**–**E**, *n* = 11–12 mice/group), with Tukey post hoc tests. Analyses made by *t* test (**F**–**J**, *n* = 5 mice/group). *P < 0.05, **P < 0.01, ***P < 0.001. Data are shown as mean ± SEM.

Given the dramatic effect of FGF21 to reduce liver triglycerides (20, 32), we were curious about the importance of sex as a biological variable for this key outcome. We measured the triglyceride content of livers collected from the mice represented in Figures 1 and 2. In agreement with the literature, we found that FGF21 treatment reduced liver triglycerides by about 70% in males, whereas it had no appreciable effect in females [*P* (treatment × sex) < 0.001; Tukey, *P* < 0.001] (Figure 3A). Because liver triglycerides of vehicle-treated males were significantly greater than those of vehicle-treated females (Tukey, *P* < 0.01), we considered the possibility of a floor effect. The data suggest this is unlikely, however, because FGF21-treated male livers had significantly *lower* triglyceride content than FGF21-treated female livers (Tukey, *P* < 0.05) (Figure 3A), indicating there was sufficient “room for improvement” despite the differing baselines. Next, we considered the possibility that female liver triglycerides were refractory to FGF21 treatment simply because this outcome may be secondary to weight loss. To explicitly isolate the contribution of weight loss, DIO male mice were assigned to 1 of 3 groups: 1 — ad libitum*–*fed + vehicle controls, 2 — ad libitum*–*fed + FGF21, and 3 — weight-matched mice, in which caloric intake was restricted to match the weight loss observed in the FGF21-treated mice (Figure 3, B–D). As expected, ad libitum*–*fed + FGF21-treated males had lower liver triglycerides compared with ad libitum*–*fed + vehicle-treated controls [ANOVA, *P* (group) < 0.01; Tukey, *P* < 0.01]. By contrast, there was no difference in the hepatic triglyceride content of ad libitum*–*fed + vehicle controls versus weight-matched mice. Last, the FGF21-treated mice had significantly less hepatic triglyceride storage than the weight-matched group (Tukey, *P* < 0.05) (Figure 3E). Next, we considered the possibility that females may decrease hepatic triglycerides in response to a higher dose of FGF21. To test this, we administered FGF21 at 1 mg/kg/d, a 5-fold higher dose than our previous experiment, to DIO female mice for 5 days. In agreement with our previous experiment (Figure 1), FGF21 did not significantly decrease body weight (Figure 3, F and G), but it significantly increased caloric intake (*t* test, *P* < 0.05) (Figure 3H). FGF21 did not alter feed efficiency (Figure 3I). Importantly, FGF21 did not decrease liver triglyceride content in DIO females, even at this higher dose (Figure 3J). Therefore, we conclude that FGF21 decreases hepatic triglycerides in a sex-dependent but weight loss–independent manner.

To identify transcriptional and metabolic pathways contributing to the sex-dependent improvement in liver triglycerides observed above, we analyzed the transcriptome and metabolome of livers collected from DIO mice after an acute treatment of FGF21 following 10 weeks on HFD. For both genes and metabolites, fold changes in response to FGF21 compared with vehicle-injected counterparts were calculated within both sexes, then tested for interaction effects between treatment and sex. Differential expression results for the treatment × sex interaction term were then mapped to Gene Ontology (GO) or Kyoto Encyclopedia of Genes and Genomes (KEGG) databases for enrichment test analyses. RNA-Seq analyses found 173 GO pathways that were significantly enriched, with the most enriched pathways being regulation of lipid metabolic process, positive regulation of mTOR signaling, response to amphetamine, white fat cell differentiation, and activin receptor signaling (Figure 3K). Metabolomic analyses found 4 significantly enriched KEGG pathways, including primary bile acid biosynthesis, beta alanine metabolism, fatty acid biosynthesis, and thyroid hormone biosynthesis (Figure 3L).

### FGF21 signaling in white adipose tissue is sex dependent.

**Figure 4:**
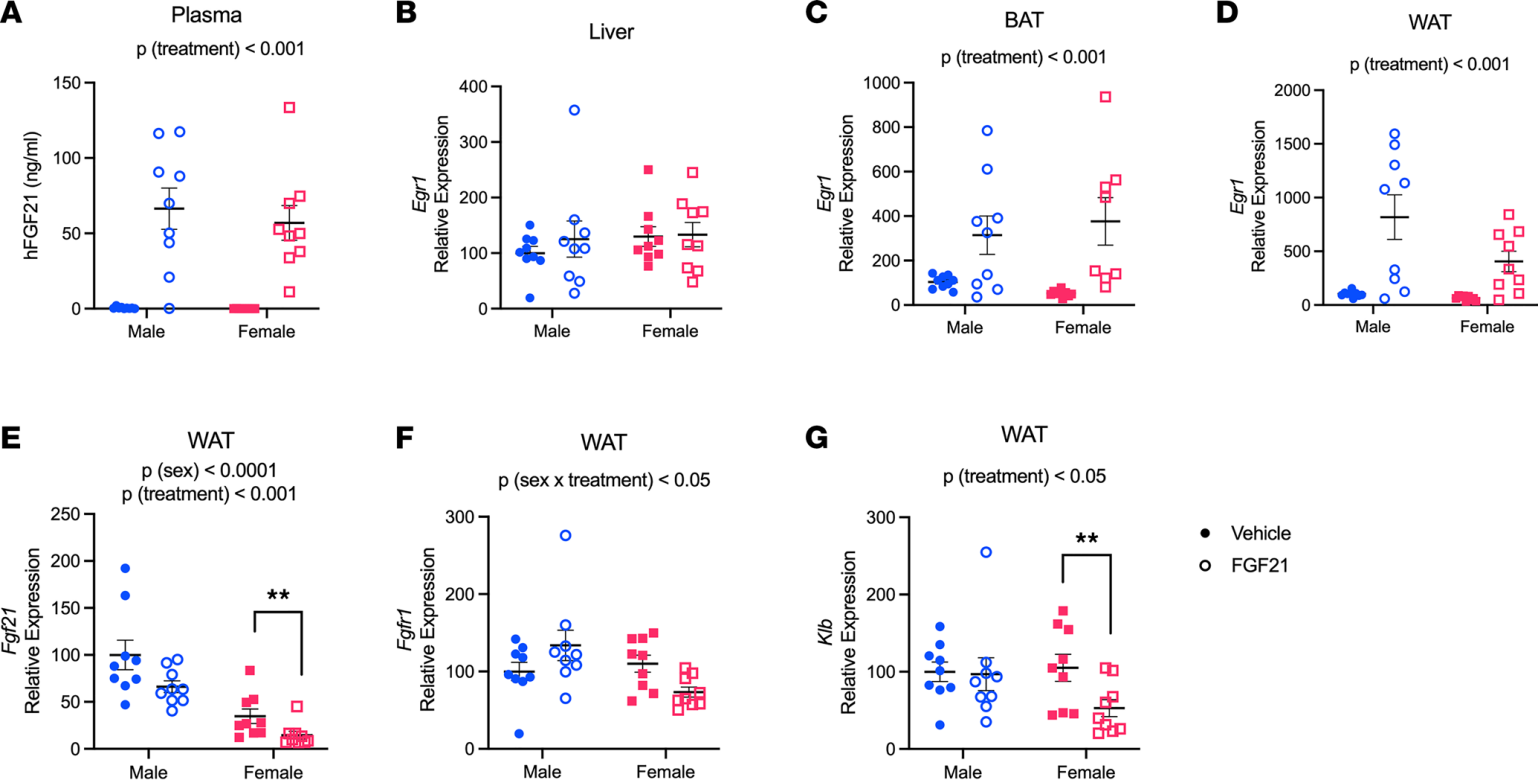
FGF21 signaling in white adipose tissue is sex dependent. At 2 hours following its i.p. injection, recombinant human FGF21 (0.1 mg/kg) was elevated in plasma collected from DIO male and female mice (**A**). We observed no effect of FGF21 on the expression of immediate early gene *Egr1* in liver (**B**). FGF21 increased *Egr1* in BAT (**C**) and WAT (**D**) of both male and female DIO mice. In WAT, endogenous expression of *Fgf21* was greater in males than females. Pharmacological injection of FGF21 reduced its endogenous expression, and this was more apparent among females (**E**). FGF21 treatment altered WAT *Fgfr1* expression in a sex-dependent manner, such that FGF21 tended to increase *Fgfr1* expression in males and decrease it in females (**F**). FGF21 decreased WAT *Klb* expression, and this effect was more apparent among females (**G**). Analyses made by 2-way ANOVA, Tukey post hoc test, **P < 0.01. Data are shown as mean ± SEM; *n* = 8–9 mice/group.

Considering the sex-dependent response to pharmacological FGF21 treatment, we explicitly addressed the influence of sex on FGF21 metabolism and on its intracellular signaling in liver and adipose tissue. First, we measured recombinant human FGF21 in plasma collected from mice 2 hours following an i.p. injection. We observed no effect of sex and no interaction between treatment and sex [*P* (treatment) < 0.001] (Figure 4A), suggesting there is no difference in its enzymatic degradation or clearance between males and females. Next, we measured the expression of the immediate early gene early growth response 1 (*Egr1*) in liver and adipose tissues. *Egr1* is induced downstream of the FGF21 receptor complex, and its expression is commonly used as a readout for successful intracellular signaling (14, 33). There was no difference in *Egr1* expression in the livers collected from FGF21- versus vehicle-treated mice of either sex, consistent with an indirect mechanism underlying FGF21’s effect on hepatic lipid metabolism (32) (Figure 4B). *Egr1* expression was greater in intrascapular brown adipose tissue (BAT) [*P* (treatment) < 0.001] (Figure 4C), and inguinal white adipose tissue (WAT) [*P* (treatment) < 0.001] (Figure 4D), of FGF21-treated mice. Again, we observed no effect of sex and no interaction between treatment and sex.

Because recent evidence supports FGF21 as a cell-autonomous regulator of adipose tissue function (34), we measured the WAT expression of FGF21 (*Fgf21*), its receptor FGF receptor 1 (*Fgfr1*), and its co-receptor β-klotho (*Klb*) in inguinal WAT. Endogenous WAT *Fgf21* expression was greater in males than females [*P* (sex) < 0.0001]. WAT *Fgf21* was reduced following treatment with recombinant FGF21 [*P* (treatment) < 0.001], and this was perhaps more apparent among females (~60% decrease in females, Tukey *P* < 0.01) (Figure 4E). We also observed a sex-dependent effect of FGF21 on its receptor(s) expression in WAT. *Fgfr1* expression was changed in a sex-dependent manner [*P* (treatment × sex) < 0.05], such that FGF21 tended to increase *Fgfr1* in males and decrease it in females (Figure 4F). *Klb* expression was decreased by FGF21 in WAT [*P* (treatment) < 0.05]. This was perhaps more apparent among females (Tukey, *P* < 0.01) (Figure 4G). Together these data support that cell-autonomous FGF21 signaling in the WAT is sex dependent.

### Adiponectin response to FGF21 is sex dependent.

**Figure 5:**
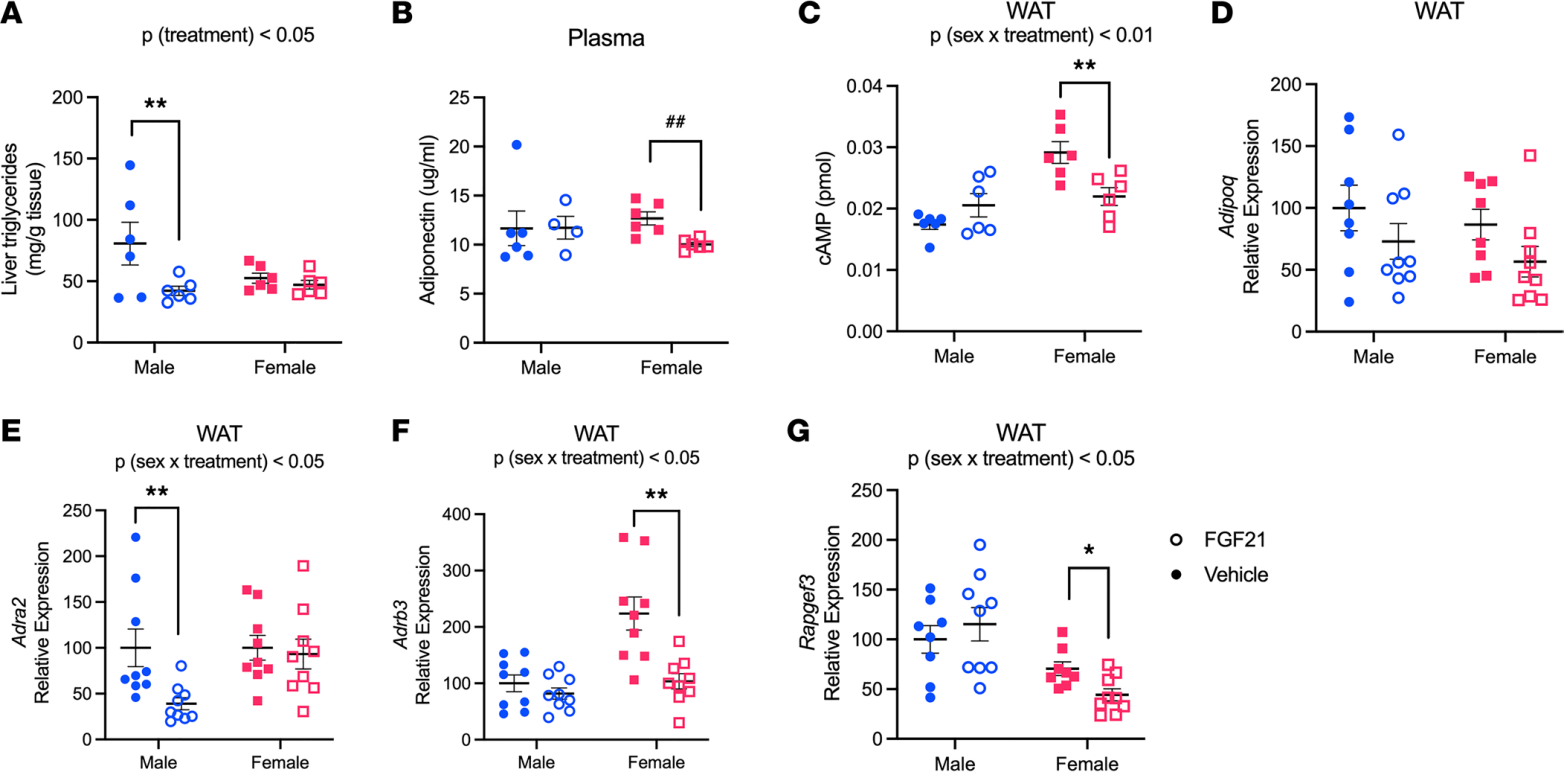
Adiponectin response to FGF21 is sex dependent. In another 12-day FGF21 administration experiment (0.1 mg/kg twice daily, i.p.), mice were euthanized 20 minutes after the final vehicle or FGF21 injection. Again, FGF21 decreased liver triglycerides in DIO male but not female mice (**A**). FGF21 decreased plasma adiponectin in females (**B**) and altered WAT cAMP in a sex-dependent manner such that FGF21 tended to increase cAMP in males and decreased it in females (**C**). Next, we measured WAT *Adipoq*, *Adra2*, *Adrb3*, and *Rapgef3* expression in tissues from mice shown in Figures 1 and 4, collected 2 hours after the final vehicle or FGF21 injection. FGF21 did not alter *Adipoq* expression (**D**) but altered *Adra2*, *Adrb3*, and *Rapgef3* expression in a sex-dependent manner (**E**–**G**). Analyses made by 2-way ANOVA, Tukey post hoc test, *P < 0.05, **P < 0.01; Mann-Whitney, ^##^*P* < 0.01. Data are shown as mean ± SEM; *n* = 6 mice/group.

**Figure 6:**
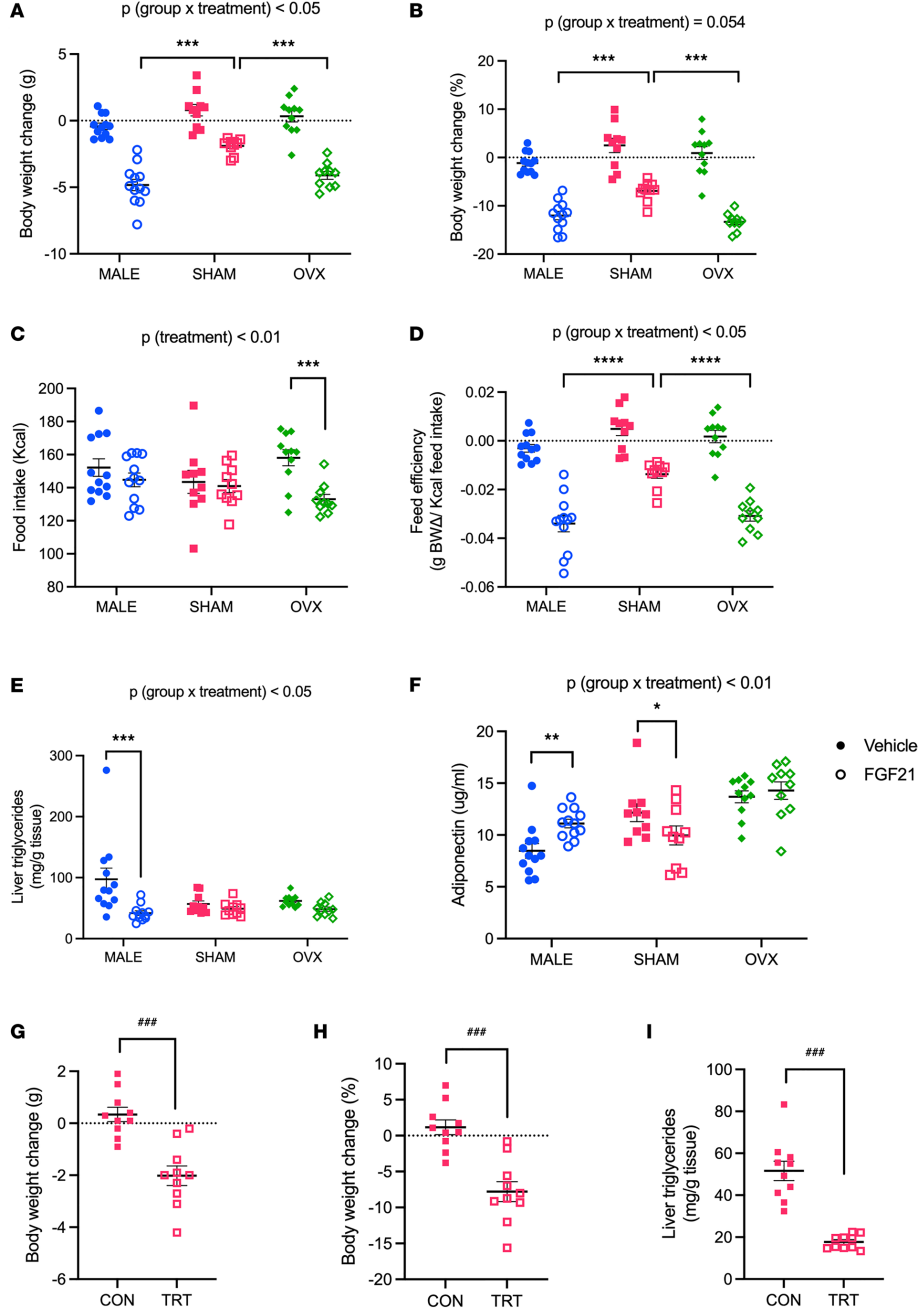
Reproductive status influences the sex-dependent control of energy balance, but not hepatosteatosis, by FGF21. In DIO male, OVX female, and sham-operated female mice, FGF21 treatment decreased body weight in a sex- and reproductive status–dependent manner, such that OVX females phenocopied the male response (**A** and **B**). FGF21 altered food intake (**C**), and feed efficiency mirrored changes in body weight change (**D**). As expected, FGF21 decreased hepatic triglycerides in males but not sham-operated females. FGF21 did not reduce liver triglycerides in OVX females despite significant FGF21-induced weight loss (**E**). Consistent with this, FGF21 altered plasma adiponectin in a sex- and reproductive status–dependent manner. FGF21 increased adiponectin in males, decreased adiponectin in sham-operated females, and had no effect in OVX females (**F**). In a separate experiment, we tested the possibility of a “floor effect” for treating hepatosteatosis in DIO females. Female mice were maintained on high-fat diet to induce obesity and hepatosteatosis. Next, half the mice were briefly switched to a chow diet. Chow diet “treatment” significantly reduced body weight (**G** and **H**) and hepatic triglycerides (**I**). Analyses made by 3-way ANOVA, Tukey post hoc test, *P < 0.05, **P < 0.01, ***P < 0.001, ****P < 0.0001 (**A**–**F**, *n* = 10–12 mice/group); *t* test, ^###^*P* < 0.001 (**E**–**G**, *n* = 10 mice/group). Data are shown as mean ± SEM.

Autocrine and/or paracrine signaling by adipocyte FGF21 promotes adipose-liver crosstalk, and reduces liver triglycerides, via the hormone adiponectin (35). Importantly, FGF21 is ineffective to reduce liver triglycerides in adiponectin-null mice, and therefore adiponectin is thought to provide a mechanism contributing to the reduction of liver triglycerides by pharmacological FGF21 treatment (17, 22). Thus, we tested the importance of sex as a variable contributing to FGF21-induced adiponectin secretion. Again, we administered FGF21 by i.p. injection to DIO male and female C57BL/6J littermates for 12 days. To confirm our previous findings, we measured liver triglycerides. We observed a significant effect of FGF21 [*P* (treatment) < 0.05]. Within males, FGF21 decreased triglyceride content by about 50% (Tukey, *P* < 0.01), whereas there was no appreciable effect within females (Figure 5A). Next, we measured circulating adiponectin, from blood collected 20 minutes after the final injection of FGF21. Among males, we observed considerable variance, and in this instance, there was no significant effect of FGF21 (but see Figure 6D). Among females, however, FGF21 unexpectedly *decreased* plasma adiponectin (Mann-Whitney, *P* < 0.01) (Figure 5B). Adiponectin exocytosis is induced downstream of the adrenergic receptor → cAMP → exchange protein directly activated by cAMP (Epac1) pathway (36). Thus, we measured cAMP in WAT collected from these mice. We observed a sex-dependent effect of FGF21 on WAT cAMP [*P* (treatment × sex) < 0.01] such that cAMP tended to be greater in FGF21-treated versus vehicle-treated males and was significantly decreased in FGF21-treated versus vehicle-treated females (Tukey, *P* < 0.01) (Figure 5C). Last, we measured mRNA for adiponectin, α_2_- and β_3_-adrenergic receptors (gene names: *Adra2* and *Adrb3*), and Epac1 (gene name: *Rapgef3*) in WAT collected from the mice in Figures 1 and 2, which were euthanized 2 hours following the final injection of FGF21. FGF21 did not alter adiponectin expression (Figure 5D). Consistent with the plasma adiponectin and cAMP data, we observed a sex-dependent effect of FGF21 treatment on adrenergic receptor and *Rapgef3* expression [*P* (treatment × sex) < 0.05]. FGF21 decreased (Gi protein–coupled) *Adra2* expression in males (Tukey, *P* < 0.01) and decreased both (Gs protein–coupled) *Adrb3* and *Rapgef3* expression in females (Tukey, *P* < 0.05) (Figure 5, E–G).

### Reproductive status influences the sex-dependent control of energy balance, but not hepatosteatosis, by FGF21.

To determine the role of the ovaries in modulating FGF21 action, we administered FGF21 for 12 days to ovariectomized (OVX) females together with sham-operated female and reproductively intact male littermates (all mice were DIO). The effect of FGF21 on body weight depended on sex and reproductive status [*P* (treatment × group) < 0.05], such that the OVX females phenocopied the male response. In this experiment FGF21 significantly reduced body weight in all 3 groups (Tukey, *P* < 0.01), but the magnitude of the effect varied according to group. Among the FGF21-treated mice, males exhibited greater weight loss compared with sham-operated females (Tukey, *P* < 0.001), and OVX females exhibited greater weight loss compared with sham-operated females (Tukey, *P* < 0.001) (Figure 6, A and B), accounting for the significant interaction term in the multivariable analysis. The effect of FGF21 on caloric intake in this experiment [*P* (treatment) < 0.01] was most apparent among OVX females (Tukey, *P* < 0.001), such that FGF21-treated OVX females consumed fewer calories than vehicle-treated controls. Within the male and sham-operated female groups, there was no significant effect of FGF21 on caloric intake (Figure 6C). This differs from the experiment shown in Figure 1; taken together these outcomes mirror the variable effect of FGF21 on caloric intake seen in the broader literature (18, 37–39). In this experiment, FGF21 significantly reduced feed efficiency in all 3 groups, but the magnitude of this effect depended on sex and reproductive status [*P* (treatment × group) < 0.05]. Among FGF21-treated mice, males had decreased feed efficiency compared with sham-operated females (Tukey, *P* < 0.0001), and OVX females had decreased feed efficiency compared with sham-operated females (Tukey, *P* < 0.0001) (Figure 6D).

The effect of FGF21 on liver triglycerides depended on sex [*P* (treatment × group) < 0.05]. FGF21-treated males had fewer liver triglycerides than vehicle-treated controls (Tukey, *P* < 0.001), whereas we again observed no decrease among sham-operated females. Despite the significant FGF21-induced weight loss, OVX females exhibited no change in hepatic triglycerides. Among the vehicle-treated mice, both sham-operated and OVX females had fewer liver triglycerides compared with males (Tukey, *P* < 0.01 and *P* < 0.05, respectively) (Figure 6E). Therefore, we performed an additional experiment to explicitly test the possibility of a “floor effect” for treating hepatic steatosis in DIO females. First, female mice were maintained on HFD for 3 weeks to induce obesity and fatty liver. Next, we switched half the females back to regular chow diet, while the others continued to eat the obesogenic diet for an additional 9 days. As a result, we observed significant weight loss (*t* test, *P* < 0.001) (Figure 6, G and H). Importantly, we observed a 66% decrease in liver triglycerides among females switched to the chow diet “treatment” (Figure 6I). Therefore, we conclude that nutritional intervention can be effective to reduce hepatic steatosis in female mice, yet FGF21 was not. Last, to determine the potential contribution of adiponectin to the phenotypes observed in FGF21-treated males and sham-operated and OVX females, we measured adiponectin in blood collected 20 minutes following the final injection. Consistent with the liver triglycerides, the effect of FGF21 on circulating adiponectin depended on sex [*P* (treatment × group) < 0.01]. In agreement with the literature, FGF21-treated males had higher plasma adiponectin than vehicle-treated controls (Tukey, *P* < 0.01). FGF21 decreased plasma adiponectin among sham-operated females (Tukey, *P* < 0.05), as in Figure 5, and there was no effect of FGF21 in the OVX group (Figure 6F).

### Aging influences the sex-dependent control of energy balance, but not hepatosteatosis, by FGF21.

**Figure 7:**
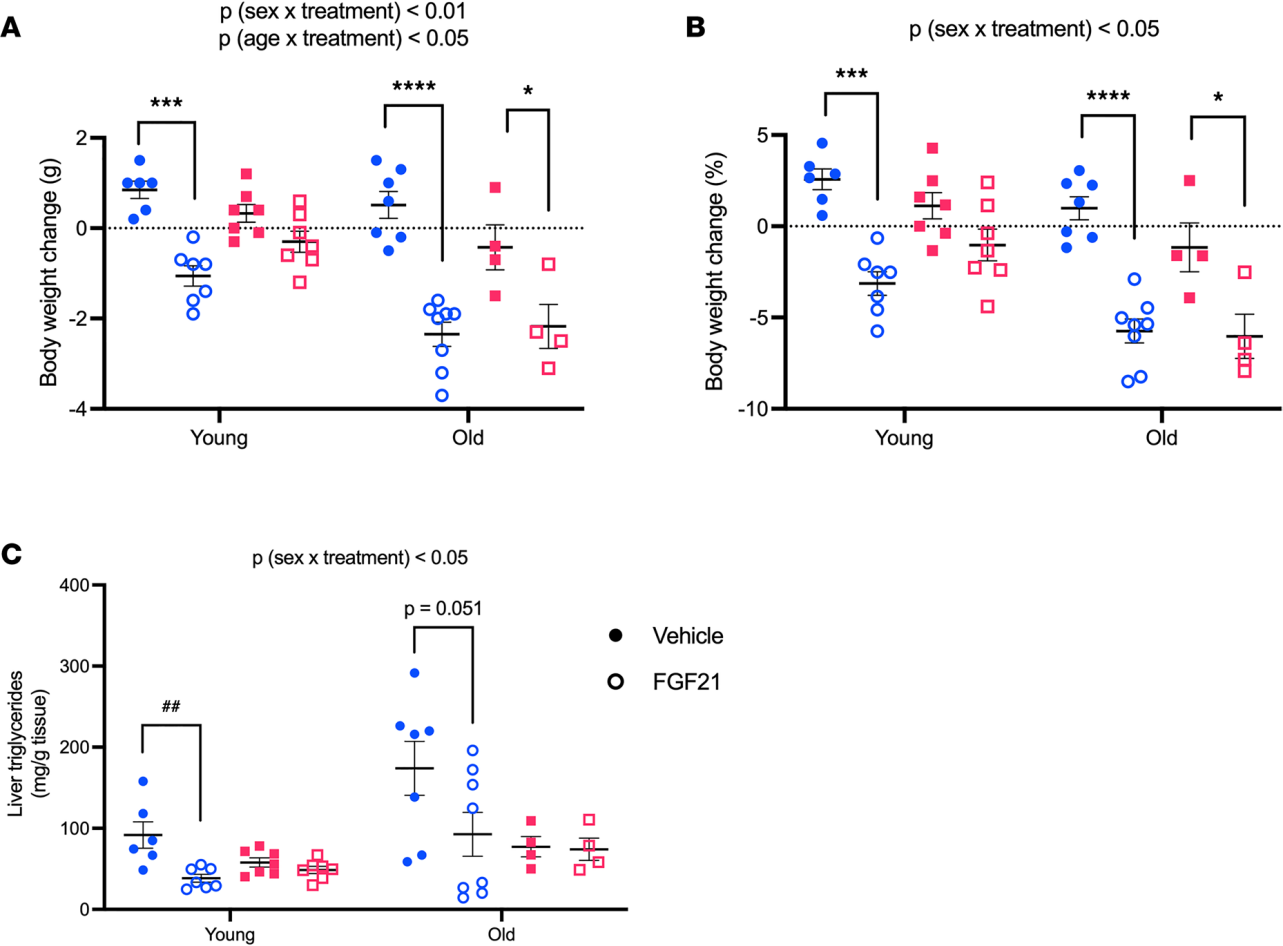
Aging influences the sex-dependent control of energy balance, but not hepatosteatosis, by FGF21. FGF21 (0.1 mg/kg twice daily, i.p., 3 days) decreased body weight in 25-month-old “old” and 4.5-month-old “young” mice in a sex- and age-dependent manner. As expected, FGF21 decreased body weight in young males but not young females. In old mice, both male and female FGF21-treated mice lost weight (**A** and **B**). By contrast, the effect of FGF21 on liver triglycerides depended on sex but not age, where FGF21-treated males decreased triglycerides while females did not (**C**). Analyses made by 3-way ANOVA, Tukey post hoc test, *P < 0.05, ***P < 0.001, ****P < 0.0001; Mann-Whitney, ^##^*P* < 0.01. Data are shown as mean ± SEM; *n* = 4–8 mice/group.

The influence of gonadal hormones is significantly diminished after reproductive senescence (40). Therefore, to further explore the role of reproductive status, we administered FGF21 for 3 days to 25-month-old “old” and 4.5-month-old “young” DIO mice, maintained on HFD for 2 weeks prior to beginning the experiment. The effect of FGF21 on body weight depended on sex and age [*P* (treatment × sex) < 0.01; *P* (treatment × age) < 0.05], such that FGF21-treated young males (Tukey, *P* < 0.001) and FGF21-treated old mice — both males (Tukey, *P* < 0.0001) and females (Tukey, *P* < 0.05) — lost more weight compared with their vehicle-treated counterparts. For both males and females, the effect of FGF21 on weight loss was greater in old versus young mice (Tukey, *P* < 0.001) (Figure 7, A and B). By contrast, the effect of FGF21 on liver triglycerides depended on sex [*P* (treatment × sex) < 0.05] but not age. Within males, FGF21-treated mice had fewer liver triglycerides than vehicle-treated controls (Tukey, *P* < 0.001). FGF21 decreased liver triglycerides among young males by approximately 60% (Mann-Whitney, *P* < 0.01) and by approximately 50% in old males (Tukey, *P* = 0.051). There was no effect of FGF21 on liver triglycerides in either young or old females, despite old females having roughly the same degree of hepatic steatosis as young males (Figure 7C).

## Discussion

In response to the sex-dependent energetic requirement for successful reproduction, natural selection favors divergent metabolic responses to nutritional challenges (1). Accordingly, males at rest tend to catabolize fatty acids for energy whereas females at rest tend to store fatty acids as triglycerides. These fat reserves are later mobilized to support successful gestation and lactation. Physiologic mechanisms directing the sex-dependent control of lipid metabolism are incompletely understood, despite important implications for reproductive and metabolic health of both women and men. The hormone FGF21 plays a critical role to direct lipid storage and utilization in both adipose tissues and liver in response to energetic and nutritional cues. We and others have recently demonstrated the sex-dependent induction of FGF21 by energetic and nutritional stressors, resulting in sex-dependent changes in energy balance (26, 31), yet whether the metabolic *response* to FGF21 is also sex dependent had not been thoroughly investigated. Altered FGF21 signaling is thought to provide a common mechanism underlying a range of metabolic effectors, including glucagon (41), PPARG agonists (8), ketogenic diet (20, 42), exercise (43), cold exposure (9), caloric and amino acid restriction (44–46), mitochondrial stress, and autophagy deficiency (47) and is itself an important therapeutic target (48, 49). Identifying the role of sex as a biological variable influencing the metabolic response to FGF21, therefore, has broad implications for the targeted application of nutritional and pharmacological treatments for metabolic disease.

In this study, we report the benefit of FGF21 treatment for improving hepatic steatosis depends on sex. In 5 independent experiments, FGF21 failed to decrease liver triglycerides in obese female mice despite an approximately 60% improvement in obese males. This does not rely on sex-dependent differences in adiposity, since we report that the effect of FGF21 to reduce liver triglycerides in males is weight loss independent. This was also not due to a floor effect since DIO female livers exhibited a robust response to a simple dietary intervention.

Surprisingly, the differential effect of sex on hepatic steatosis did not depend on the presence of an intact female reproductive system, nor did it diminish with age. Both OVX and aged female livers remain refractory to the triglyceride-reducing benefits of FGF21. These findings suggest the observed sex differences do not depend on current ovarian hormone levels. That is, ovarian hormones do not have an “activational” role (50, 51) to direct the sex-dependent hepatic response to FGF21. This result agrees with a recent mechanistic account of sexually divergent liver metabolic strategies, wherein estrogen receptor α plays a key “organizational” role to direct fuel selection and lipid handling that is imprinted around the time of parturition (52). That is, estrogen signaling in liver is critical to establish its notably sex-dependent strategies for lipid handling, but the sensitive period for this divergence occurs very early in life and does not rely on the presence of ovarian hormones in adults (52).

To identify liver metabolic pathways associated with the sex-dependent improvement in liver triglycerides observed above, we analyzed the sex-dependent transcriptomic and metabolomic response in livers collected from DIO mice acutely treated with FGF21 or vehicle. The most significantly enriched GO pathway for the treatment × sex interaction term was regulation of lipid metabolic process, which included differential expression of PPARG and several of its lipogenic target genes (Supplemental Figure 1; supplemental material available online with this article; https://doi.org/10.1172/jci.insight.155848DS1). Accordingly, white fat cell differentiation, a lipogenic pathway, and positive regulation of mTOR signaling, which activates PPARG and downstream lipogenic processes in liver (53, 54), were also among the top 5 significantly enriched pathways in this unbiased analysis. In agreement with the transcriptomics, and with the sex-dependent effect on hepatic steatosis, “fatty acid biosynthesis” was among the 4 differently enriched KEGG pathways identified in our metabolomic analysis (Supplemental Figure 2). Intriguingly, the activin receptor signaling pathway also appeared among the top 5 most significantly enriched GO pathways. Accumulating evidence points to a complex role for activins, and their natural inhibitor follistatin, to modulate hepatic lipid accumulation and fibrosis in cells, rodents, and humans (55, 56), and to the modulation of this pathway by adiponectin (57, 58), suggesting a potentially novel pathway by which FGF21 may alter liver triglycerides in a sex-dependent manner.

Consistent with the observed sex-dependent hepatic response to FGF21 treatment, we also observed a sex-dependent effect of FGF21 to induce adiponectin secretion from WAT, via adrenergic receptor signaling, cAMP, and Epac1. The mechanism(s) by which FGF21 improves hepatic steatosis are incompletely understood, but the pathway does not appear to be autocrine or paracrine because *Fgfr1* is only sparsely expressed in hepatocytes (59) and because *Klb*-null and wildtype livers are equally responsive to FGF21 (32). Rather, the reduction in liver triglycerides depends on interorgan crosstalk. The hormone adiponectin is secreted from WAT and from 3T3-L1 cells following FGF21 treatment, and several of the metabolic benefits of FGF21 are thought to depend on adiponectin signaling (16, 17). Specifically, FGF21 treatment reduces liver triglycerides by about 50% in wildtype male mice while this is abrogated in adiponectin-null males (17).

Here, we found that FGF21 increased plasma adiponectin in males while reducing it in females and having no significant effect in OVX mice. Adiponectin exocytosis from white adipocytes is stimulated by adrenergic receptor signaling and cAMP (60, 61), via increased expression of *Epac1* (36). Accordingly, we observed a sex-dependent effect of FGF21 on adrenergic receptor expression. mRNA for the Gi-coupled α_2_-adrenergic receptor was decreased in males but not females, whereas Gs-coupled β_3_-adrenergic receptor mRNA was increased. Such a change would be expected to increase cAMP in males, because of reduced inhibitory signaling, and decrease cAMP in females, because of reduced excitatory signaling. Indeed, this is what we observed. Both cAMP and its downstream target *Epac1* tended to be increased by FGF21 in males and were reduced by FGF21 in females. While these findings are consistent with sex-dependent effects of FGF21 on adiponectin exocytosis, they do not measure its secretion directly, nor do they rule out the potential contribution of differential clearance.

In agreement with a recently described feed-forward regulatory loop, locating plasma adiponectin downstream of autocrine *Fgf21* expressed by adipocytes (35), we also found the expression of WAT *Fgf21* was diminished by pharmacological FGF21 treatment in females. Likewise, its receptor complex (*Fgfr1* and *Klb*) responded to FGF21 treatment in a sex-dependent manner. FGFRs’ expression was diminished in WAT collected from DIO, FGF21-treated females compared with vehicle-treated female littermates. To the extent the metabolic effects of FGF21 vary according to FGFR expression (62, 63; but see ref. 64), these findings suggest the possibility that FGF21 induces an “FGF21-resistant” (65) state in female WAT, thereby preserving lipid storage during nutritional stressors.

In addition to improving hepatic steatosis in males, FGF21 decreases body weight and total body fat by increasing energy expenditure (38, 66). Here we report the effect of FGF21 on body weight, adiposity, and feed efficiency depends on sex. The findings are consistent with our recent observation that metabolic responses to dietary protein “dilution” (67–69) are sex dependent (31). FGF21 is secreted from the livers of rodents and humans in response to amino acid restriction and macronutrient imbalance (6, 30) and is required for the increase in energy expenditure and weight loss (68) observed in males maintained on low–amino acid or low-protein diets. In our hands, body weight loss in response to protein dilution was abrogated in females and this was reversed by ovariectomy (31). In the current study, the ability of pharmacological FGF21 treatment to reduce body weight depended on sex, such that females lost relatively less weight compared with males; the magnitude of this sex difference varied between experiments (see Figures 1, 6, and 7). This contrasts with a recent publication (70) reporting that body weight and fat loss following FGF21 administration is independent of sex in C57BL6/J mice. Possible explanations for this discrepancy include differences in environmental stress during the experiments, since the saline-injected mice in that study lost approximately 7% body weight over the course of the experiment whereas control groups in the present study maintained their body weights. More likely, the different findings arise from differences in statistical power to detect a significant interaction term in the ANOVA, since the *n* size for each treatment group was roughly double in the present study. By contrast, a very recent study by the same group (71), in this case using the agouti mouse model of melanocortin dysfunction, found that FGF21-treated females lost neither body weight nor liver triglycerides. Underlying mechanisms, including the importance of sex hormones, were not identified. In our hands, the sex-dependent body weight response to FGF21 was reversed by ovariectomy and by advanced age. Therefore, the sexually divergent effect of FGF21 on energy balance likely depends on current levels of ovarian hormones, though downstream effectors remain undetermined.

Not all the metabolic responses to FGF21 treatment in this study were sex dependent. FGF21 robustly improves glucose tolerance in rodents, though its clinical efficacy for this endpoint has largely been disappointing (72, 73). The translational discrepancy likely arises because the glucose-lowering effects of FGF21 depend on its action in BAT and require uncoupling protein 1 (13, 74) and because BAT has an overall larger impact on adult rodent physiology compared with humans (75). Here, we found FGFR signaling in BAT, measured by *Egr1*, was significantly increased following FGF21 treatment in both females and males. Accordingly, FGF21 improved glucose tolerance in both sexes.

There are limitations to this study that could be addressed in future research. First, the current studies were entirely pharmacological. The extent to which differential actions of endogenously produced FGF21 directs sex differences in hepatic lipid handling in response to a short fast (5), macronutrient imbalance (30), or pregnancy and lactation (76) remains undetermined. Likewise, differential endogenous FGF21 action is thought to provide a mechanism for a number of upstream metabolic manipulations (discussed above, refs. 8, 9, 20, 41–46), and the extent to which sex-dependent FGF21 action influences these responses is largely undetermined. Moreover, the current studies used only 2 doses of recombinant FGF21 delivered over a limited time course. Within this framework, our findings clearly support the differential sensitivity of male and female livers. Additional dose-response studies are needed to better inform the targeted delivery of FGF21-based therapeutics. Our findings suggest that sex and reproductive status are key biological variables influencing efficacy for some but not all metabolic endpoints. We report sex-dependent responses for FGF21-induced body weight and fat loss and for feed efficiency; further studies will be required to directly test differences in energy expenditure. Last, the current studies use a single rodent model of diet-induced obesity. Additional analysis of clinical data (21, 77), disaggregating the data by sex and reproductive status as suggested (78), is particularly important for clinical studies of liver disease (79) and can begin to determine the translational relevance of our findings. One potential revelation of the more nuanced analysis we propose, for example, is that FGF21-based therapies could be substantially more effective in men than is currently appreciated, but this efficacy is obscured by a less robust response from the women.

Here we identified a key role for sex as a biological variable to direct the hepatic response to FGF21, via sex-dependent induction of the adrenergic receptor → cAMP → Epac1 → adiponectin pathway. In agreement with this, the differential effect of FGF21 on liver included sex-dependent activity in lipid metabolic, mTOR, and activin signaling pathways. The sex-dependent effect on liver triglycerides did not depend on an intact female reproductive system, nor did it diminish with advanced age. Because FGF21 provides a downstream mechanism contributing to several metabolic interventions and given its direct clinical application for the treatment of fatty liver and other metabolic diseases, these findings may have broad implications for the targeted application of nutritional and pharmacological treatments for metabolic disease.

## Methods

### Animals.

Age-matched male and female C57BL/6J wildtype mice were obtained from The Jackson Laboratory or bred in-house. Mice were single-housed on a 12-hour light/12-hour dark cycle in a temperature- (20°C to 22°C) and humidity-controlled vivarium. Where indicated, DIO mice were placed on 60% HFD (Research Diets, catalog D12492) for 5–6 weeks, unless otherwise specified, to induce obesity and hepatosteatosis before undergoing experiment. Mice were 9–14 weeks old when placed on HFD, except for in the aging experiment, where “old” mice were 25 months old. In all experiments, mice were counterbalanced into treatment groups based on body weight. Mice had ad libitum access to food and water unless otherwise specified.

### FGF21 administration.

Recombinant human FGF21 (ProSpecBio, catalog CYT-474) was first dissolved in water according to the manufacturer’s instructions, then further diluted in 0.9% sterile saline for injection. For chronic experiments, FGF21 was administered for 3, 5, or 12 consecutive days as indicated. For 3- and 12-day experiments, FGF21 was given at a dose of 0.1 mg/kg twice daily i.p., just after onset of the light phase and again just before onset of the dark phase. For the high-dose experiment, FGF21 was administered for 5 days at 1 mg/kg once daily subcutaneously, just before onset of the dark phase. Body weight and food intake were measured daily. For the transcriptomic and metabolomic endpoints, we chose an acute time course to isolate the immediate effects of FGF21 on liver, rather than changes that may be secondary to its systemic effects on body weight and adiposity. We gave 1 dose of FGF21 (0.1 mg/kg, i.p.) just before the onset of the dark phase, and another dose (0.1 mg/kg, i.p.) was given after the onset of the light phase the following day; animals were euthanized 2 hours following the morning injection.

### Body composition.

Body composition was measured by time domain NMR using a Minispec LF110 body composition analyzer (Bruker) on day –2 and day 7 of a 12-day chronic FGF21 injection experiment.

### Glucose tolerance test.

Glucose tolerance tests were performed on day 0 and day 10 of a 12-day chronic FGF21 injection experiment. Blood glucose was measured from the tail vein (Accu-Check glucometer and Aviva strips; Roche Diagnostics) immediately following the morning dose of FGF21 or vehicle. Two hours later, blood glucose was again measured immediately prior to a dextrose administration (2 g/kg, i.p.) and at 15, 30, 60, and 120 minutes postinjection.

### Weight match experiment.

Male mice were placed on HFD for 5 weeks, then counterbalanced by body weight into 3 groups: 1 — ad libitum–fed + vehicle controls, 2 — ad libitum–fed + FGF21, or 3 — weight-matched. In the weight-matched group, baseline food intake was measured from day –2 to day 0, and then food was restricted on experiment days to 70%–80% of their baseline intake to match the weight loss observed in the FGF21-treated group. The experiment lasted 12 days, and body weight and food intake were measured daily. All mice remained on HFD throughout the study.

### Ovariectomy.

Female mice were placed on HFD for 5 weeks, then counterbalanced by body weight into OVX or sham group prior to undergoing surgery. Briefly, mice were anesthetized under isoflurane, and then a midline incision of approximately 1 cm was made at the mid-dorsum of the mouse. Forceps were used to gently pull apart the ovary and fat pad off the uterine horn, the ovary was excised, and the uterine horn was replaced in the abdominal cavity. The procedure was repeated to remove the second ovary, and then the incision was closed. Sham procedures were performed by making the incision as described but without removing any tissue. Animals were monitored daily for 7 days postoperatively, then were allowed to recover for an additional week before undergoing experiments. Male mice were placed on HFD at the same time as female mice but did not undergo any surgical procedures.

### Plasma analyses.

Whole blood was collected from trunk blood at euthanasia into chilled EDTA-coated tubes, then centrifuged at 3,000*g* for 30 minutes at 4°C. Plasma was stored at –80°C for later analysis. FGF21 was measured using a commercially available kit (MilliporeSigma, catalog EZRMFGF21-26K) following the manufacturer’s instructions. Adiponectin was also measured using a commercially available kit (MilliporeSigma, catalog EZMADP-60K) following the manufacturer’s instructions.

### Hepatic triglyceride content.

Livers were collected at euthanasia, snap-frozen in liquid nitrogen, and stored at –80°C for later analysis. Briefly, a piece of liver was placed in a volume of Tris buffer (50 mM Tris, 150 mM NaCl, 1 mM EDTA, pH 7.5) scaled to weight at 1 mL/100 mg of tissue in reinforced tubes with 2 stainless steel beads, then homogenized for 20 seconds using a bead beater (Mini-Beadbeater-16, BioSpec Products). Homogenized samples were rested on ice for 1.5 hours, then incubated in a 96-well plate in a 1:100 ratio with Triglycerides Liquid Reagent Set (Pointe Scientific, catalog T7532) at 37°C for 5 minutes. Samples were analyzed in a microplate spectrophotometer (BioTek, Epoch 2) at 500 nm. Sample concentrations were calculated from a standard curve created using Triglyceride GPO Standard (Pointe Scientific, catalog T7531-STD).

### cAMP quantification.

WAT was collected at euthanasia, snap-frozen in liquid nitrogen, and stored at –80°C for later analysis. cAMP was quantified using a commercially available kit (Abcam, catalog ab65355) following the manufacturer’s instructions, including an optional deproteinization step using an additional commercially available kit (Abcam, catalog ab204708).

### Gene expression analysis.

Tissues were collected at euthanasia and frozen in liquid nitrogen (liver, WAT) or 2-methylbutane on dry ice (BAT) and stored at –80°C for later analysis. Total RNA was isolated from tissues using the RNeasy Mini Kit (QIAGEN, catalog 74106) according to the manufacturer’s instructions. cDNA was synthesized using the High-Capacity cDNA Reverse Transcription Kit (Thermo Fisher Scientific, catalog 4368814). Gene expression analysis was conducted using TaqMan gene expression assays (Thermo Fisher Scientific) and analyzed on a Bio-Rad CFX384. Expression of adiponectin (*Adipoq*; Mm00456425_m1), α_2_-adrenergic receptor (*Adra2a*; Mm00845383_s1), β_3_-adrenergic receptor (*Adrb3*; Mm02601819_g1), β-klotho (*Klb*; Mm00473122_m1), early growth response 1 (*Egr1*; Mm00656724_m1), fibroblast growth factor receptor 1 (*Fgfr1*; Mm00438930_m1), and fibroblast growth factor 21 (*Fgf21*; Mm00840165_g1) was normalized to the housekeeping genes ribosomal protein lateral stalk subunit P0 (*Rplp0*; Mm00725448_s1), 18S rRNA (*Rn18s*; Mm04277571_s1), or peptidylprolyl isomerase B (*Ppib*; Mmm00478295_m1) using the 2^-ΔΔCt^ method.

### Transcriptomic data acquisition and analyses.

Total RNA was isolated from tissues using the RNeasy Mini Kit (QIAGEN, catalog 74106) according to the manufacturer’s instructions. Gene expression profiling was carried out using a 3′-Tag-RNA-Seq protocol. Barcoded sequencing libraries were prepared using the QuantSeq FWD kit (Lexogen) for multiplexed sequencing according to the recommendations of the manufacturer using both the UDI adapter and UMI Second Strand Synthesis modules (Lexogen). The fragment size distribution of the libraries was verified via microcapillary gel electrophoresis on a LabChip GX system (PerkinElmer). The libraries were quantified by fluorometry on a Qubit fluorometer (Life Technologies) and pooled in equimolar ratios. The library pool was Exonuclease VII (New England BioLabs) treated, solid phase reversible immobilization bead purified with KapaPure beads (Kapa Biosystems, Roche), and quantified via quantitative PCR with a Kapa Library Quantification Kit (Kapa Biosystems, Roche) on a QuantStudio 5 RT-PCR system (Applied Biosystems). Up to 48 libraries were sequenced per lane on a HiSeq 4000 sequencer (Illumina) with single-end 100 bp reads. Quality of raw reads was assessed using fastqc (https://www.bioinformatics.babraham.ac.uk/projects/fastqc/). rRNA content was determined by aligning the reads against a set of *Mus musculus* rRNA sequences using bowtie2 v.2.3.4.1 (http://bowtie-bio.sourceforge.net/index.shtml). Raw reads were processed and trimmed to removed phiX and adapter sequences and to remove the first 11 bases from each read. Processed reads were aligned to GRCm38 primary genome assembly using GENCODE vM15 annotation, with STAR v.2.6.0c (80), to generate counts per gene. Tables of raw counts and counts per million (CPM) normalized counts were generated. Prior to analysis, genes with low expression (defined here as those with mean CPM less than 0.5) were filtered, leaving 13,180 genes. Differential expression analyses were conducted using the limma-voom Bioconductor pipeline (http://www.bioconductor.org/packages/release/bioc/html/limma.html), using a model that included effects for treatment group, sex, and the treatment group × sex interaction. Statistical analyses were conducted by the University of California, Davis, Genome Center Bioinformatics Core. Data have been deposited in NCBI’s Gene Expression Omnibus (GEO) (81) and are accessible through GEO Series accession number GSE210683 (https://www.ncbi.nlm.nih.gov/geo/query/acc.cgi?acc=GSE210683).

### Metabolomic data acquisition and analyses.

Frozen livers were submitted to the West Coast Metabolomics Center at the University of California, Davis, for untargeted primary metabolism analysis by automated liner exchange cold injection system gas chromatography time-of-flight mass spectrometry (MS). Data were acquired as previously described (82). Briefly, Restek Corporation Rtx-5Sil MS columns were used with helium as the mobile phase. Column temperature was 50°C–330°C, flow rate was 1 mL/min, and injection volume was 0.5 μL (injected 25 splitless time into a multibaffled glass liner). Injection temperature was 50°C ramped to 250°C by 12°C/s. Oven temperature program was 50°C for 1 minute, then ramped at 20°C/min to 330°C, held constant for 5 minutes. MS parameters were as follows: Leco Pegasus IV mass spectrometer used with unit mass resolution at 17 spectra/s from 80–500 Da at –70 eV ionization energy and 1800 V detector voltage with a 230°C transfer line and a 250°C ion source. ChromaTOF (vs 2.32) was used for data analysis and review of chromatograms. Metabolomics data were log_2_ transformed and cyclic loess normalized using the function normalizeCyclicLoess in the limma Bioconductor package, then analyzed in limma as detailed for the RNA-Seq analysis. Statistical analyses were conducted by the University of California, Davis, Genome Center Bioinformatics Core. This study is available at the NIH Common Fund’s National Metabolomics Data Repository website, the Metabolomics Workbench (83), https://www.metabolomicsworkbench.org, where it has been assigned Study ID ST002249. The data can be accessed directly via their Project DOI: http://dx.doi.org/10.21228/M8ZX31 This work is supported by NIH grant U2C-DK119886.

### Statistics.

The remaining data were analyzed using SigmaPlot and GraphPad Prism by 1-, 2-, or 3-way ANOVA or 2-tailed Mann-Whitney *U* test or *t* test, as indicated in figure legends. Multiple comparisons were made using Tukey post hoc tests. A *P* value less than 0.05 was considered significant. Graphs were created using GraphPad Prism. Data are presented as means ± SEM unless otherwise noted.

### Study approval.

All animal experiments were approved by the Institutional Care and Use Committee of the University of California, Davis.

## Author contributions

ATC designed, conducted, and analyzed experiments and wrote the manuscript. KRL designed, conducted, and analyzed experiments and edited the manuscript. KPH, CTW, NG, YF, DJ, KASS, LCS, NM, and MLG conducted experiments and edited the manuscript. KKR conceptualized and designed the study, executed experiments, analyzed and interpreted data, and wrote the manuscript.

## Supplementary Material

Supplemental data

## Version 1 08/23/2022

In-Press Preview

## Version 2 10/10/2022

Electronic publication
