# Supplementary material for: FGF21 controls hepatic lipid metabolism via sex-dependent interorgan crosstalk
Source: JCI Insight. 2022 Oct 10;7(19):e155848. doi: 10.1172/jci.insight.155848 (PMC9675565; doi:10.1172/jci.insight.155848)
Supplement: Supplemental data [file jciinsight-7-155848-s008.pdf]

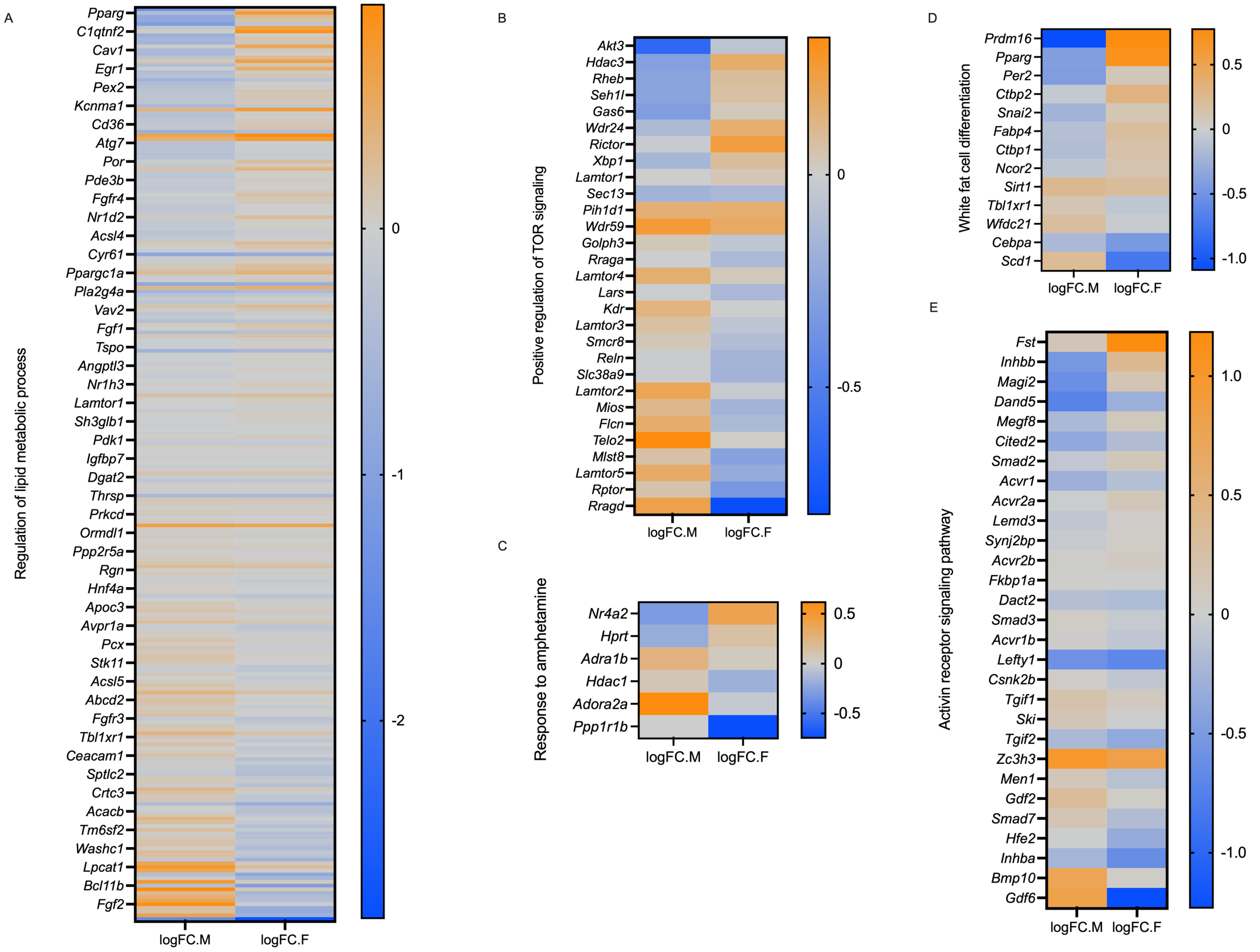

Supplemental Figure 1. Transcriptomic analysis of male and female livers in response to FGF21. Top 5 of 173 significantly enriched GO pathways for the (treatment x sex) interaction term were: “regulation of lipid metabolic process” (A), “positive regulation of TOR signaling” (B), “response to amphetamine” (C), “white fat cell differentiation” (D), and “activin receptor signaling pathway” (E). Color indicates fold change of genes in FGF21-treated animals compared to vehicle-treated controls in males (logFC.M) next to females (logFC.F) within each pathway.

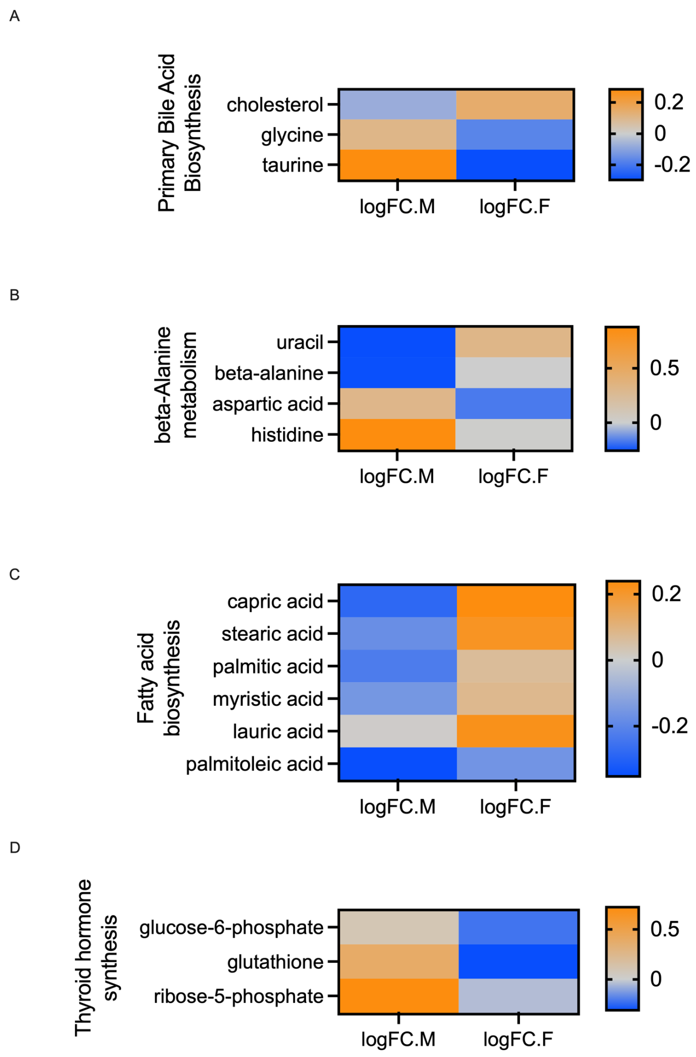

Supplemental Figure 2. Metabolomic analysis of male and female livers in response to FGF21. Four KEGG pathways were significantly enriched for the (treatment x sex) interaction term: “primary bile acid biosynthesis” (A), “beta-Alanine metabolism” (B), “fatty acid biosynthesis” (C), and “thyroid hormone synthesis” (D). Color indicates fold change of metabolites in FGF21-treated animals compared to vehicle-treated controls in males (logFC.M) next to females (logFC.F) within each pathway.
